# Transplant Trial Watch

**DOI:** 10.3389/ti.2024.13457

**Published:** 2024-07-22

**Authors:** John M. O’Callaghan, Simon Knight, John Fallon

**Affiliations:** ^1^ University Hospitals Coventry and Warwickshire, Coventry, United Kingdom; ^2^ Centre for Evidence in Transplantation, Nuffield Department of Surgical Sciences, University of Oxford, Oxford, United Kingdom; ^3^ Oxford Transplant Centre, Churchill Hospital, Oxford, United Kingdom

**Keywords:** kidney transplantation, living donor liver transplantation, randomised controlled trial, acute kidney injury, tacrolimus

To keep the transplantation community informed about recently published level 1 evidence in organ transplantation ESOT and the Centre for Evidence in Transplantation have developed the Transplant Trial Watch. The Transplant Trial Watch is a monthly overview of 10 new randomised controlled trials (RCTs) and systematic reviews. This page of Transplant International offers commentaries on methodological issues and clinical implications on two articles of particular interest from the CET Transplant Trial Watch monthly selection. For all high quality evidence in solid organ transplantation, visit the Transplant Library: www.transplantlibrary.com.

Randomised Controlled Trial 1 Fixed Low Dose Versus Concentration-Controlled Initial Tacrolimus Dosing With Reduced Target Levels in the Course After Kidney Transplantation: Results From A Prospective Randomized Controlled Non-Inferiority Trial (Slow and Low study).

*by Stumpf, J., et al. EClinicalMedicine 2024; 67: 102381.*

## Aims

To assess if a slow and low tacrolimus regimen is non-inferior to classical dose of tacrolimus with regards biopsy proven acute rejection (BPAR) in an adult kidney transplant population.

## Interventions

Participants were randomised to receive standard of care which was basiliximab induction, MMF, steroids and tacrolimus with trough levels 7–9 mg/mL or “slow and low” regimen of basiliximab induction, MMF, steroids and tacrolimus with 5 mg/day fixed for 7 days when to a trough level of 5–7 ng/mL.

## Participants

432 adult kidney transplant recipients receiving ABO-compatible organs with low immunological risk scores, from living or deceased donors.

## Outcomes

Primary efficacy outcome was the combined endpoint of BPAR, graft failure and death within 6 months. Secondary endpoints were renal function, delayed graft function. Chronic ABMR, DSAs, PTDM, infective incidence.

## Follow-Up

6 months post-transplantation.

## CET Conclusion

*by John Fallon*

This large multi-centre European open-label RCT demonstrated non-inferiority of a slow and low tacrolimus regimen with regards their composite end-point of BPAR, graft failure and death over a period of 6 months. However, one should be cautious in the interpretation. It is important to note that the study was conducted in immunologically low risk recipients, clearly recipients with a negative CDC cross-match, but also no history of rejection in previous allografts, no DSAs, PRA <20% and no DCD organs, which in a wider context does limit the impact of the regimen’s presented non-inferiority. When scrutinising the results more closely, the combined primary end-point occurred in 20.3% of slow and low and 18.8% of the standard care, risk difference and two-sided 90% confidence interval 1.5% (−6.0%; 9.0%; one-sided test of equivalence with a non-inferiority margin of 12.5% *p* = 0.008), but in this context a non-inferiority margin of 12.5% could be considered too large, but if reduced to margins closer to 5%, which one might consider more appropriate in this context, significance would likely not be reached. This combined with the finding that there was a statistically higher percentage of BANFF IA-III, i.e., above borderline, in the slow and low regimen compared with standard (11.6% vs. 5.2%, *p* = 0.027) could be a concern. The assessment on the impact of these is limited by the duration of follow-up being only 6 months, given these subtle event changes are impactful on the ultimate lifespan of a graft rather than necessarily acute losses. We must then consider conceptually the overall reason for interest in a slow and low regimen, which is the effects of early high trough levels. Slow and low avoided concerningly high trough levels within the first week, and by week 4 the levels in standard and slow and low are equilibrated, with acceptable therapeutic levels for nearly all patients throughout. However, despite this no difference was observed in secondary outcome parameter such as AE, SAE, kidney function, neurotoxicity, PTDM, or DGF (the study duration being too limited to consider implication to cardiovascular risk factors). While standardising early tacrolimus use is attractive for its clinical ease and its potential non-inferiority to standard care, the fact remains that variations in tacrolimus metabolism exist, and the present study is insufficient to confidently demonstrate the non-inferiority or reasoning behind a slow and low regimen.

## Jadad Score

3.

## Data Analysis

Modified intention-to-treat analysis.

## Allocation Concealment

Yes.

## Trial Registration

EudraCT—2013-001770-19.

## Funding Source

Industry funded.

**Randomised Controlled Trial 2**

Effect of Dexmedetomidine on the Incidence of Postoperative Acute Kidney Injury in Living Donor Liver Transplantation Recipients: A Randomized Controlled Trial.

*by Kwon, H. M., et al. International Journal of Surgery 2024 [record in progress].*

## Aims

The aim of this study was to investigate the role of intraoperative dexmedetomidine infusion on the incidence of acute kidney injury (AKI) in living donor liver transplant patients.

## Interventions

Participants were randomised to receive either an infusion of dexmedetomidine or 0.9% saline.

## Participants

214 living donor liver transplant patients.

## Outcomes

The primary endpoint was the incidence of AKI. The secondary endpoints were levels of serial lactate during surgery, overall mortality, graft failure, early allograft dysfunction, major adverse cardiovascular events, chronic kidney disease, duration of mechanical ventilation, intensive care unit (ICU) and hospital length of stay.

## Follow-Up

3 months posttransplantation.

## CET Conclusion

*by Simon Knight*

This interesting paper from a single centre in South Korea investigated the use of dexmedetomidine (an alpha-2 agonist with anti-inflammatory and anti-oxidant properties) as a renoprotective agent during living-donor liver transplantation. 205 recipients were randomised to dexmedetomidine or control (saline) infusion during surgery. The authors report a significant reduction in risk of acute kidney injury in the dexmedetomidine group (35% vs. 50%), with lower postreperfusion lactate levels, although no difference in incidence of post-reperfusion syndrome. The study appears well designed, with adequate randomisation, allocation concealment and double-blinding. The exact method by which the clinical team were blinded to intervention is unclear—placebo was used, but how this was masked was not described. Given the evidence available from this study and others in cardiac surgery, it certainly warrants further investigation in more mixed multicentre cohorts.

## Jadad Score

4.

## Data Analysis

Per protocol analysis.

## Allocation Concealment

Yes.

## Trial Registration

ClinicalTrials.gov—NCT03522688.

## Funding Source

Non-industry funded.

## Clinical Impact Summary

*by John O’Callaghan*

This is an interesting study in living donor liver transplantation. The trial was conducted as a randomised, double-blind and placebo-controlled trial. The randomisation was computer generated and kept in sealed envelopes, opened prior to surgery by the anaesthetic nurse. AKI was defined using the KDIGO guidelines up to 7 days after surgery. There were very few dropouts and no group crossovers. The power calculation was based on the groups’ previous work, where the risk of AKI was 59%. Altogether the setup, design and conduct of the trial is good.

The results showed a significant reduction in AKI when dexmedetomedine was used (35% versus 50%) and lower serum lactate levels until the end of surgery. There was no significant difference in CKD, MACE or EAD. There was no significant difference in ICU or hospital stay.

The majority of the reduction in AKI risk was seen in those with only stage 1 AKI (28% versus 38%). There was a moderate reduction in stage 2 AKI (6% versus 11%), but this was not statistically assessed, and no difference in the small risk of stage 3 AKI (1%). Therefore a far larger study would be required to demonstrate any difference in stage 2 or 3 AKI, and much longer follow up to establish if there are any consequences of the modest reduction in stage 1 AKI. Another option is to focus on patients with pre-existing CKD, who may benefit more from any protective effect.

